# Why Are Some Listeria monocytogenes Genotypes More Likely To Cause Invasive (Brain, Placental) Infection?

**DOI:** 10.1128/mBio.03126-20

**Published:** 2020-12-15

**Authors:** José A. Vázquez-Boland, Martin Wagner, Mariela Scortti

**Affiliations:** a Microbial Pathogenesis Laboratory, Infection Medicine, Edinburgh Medical School (Biomedical Sciences), University of Edinburgh, Edinburgh, United Kingdom; b Institute of Food Safety, Food Technology and Veterinary Public Health, University of Veterinary Medicine, Vienna, Austria; University of British Columbia

**Keywords:** *Listeria monocytogenes*, virulence heterogeneity, hypervirulent strains, prolonged *in vivo* survival, invasive listeriosis, *in vivo* survival, maternal-fetal listeriosis, neurolisteriosis

## Abstract

Although all isolates of the foodborne pathogen Listeria monocytogenes are considered to be pathogenic, epidemiological evidence indicates that certain serovar 4b lineages are more likely to cause severe invasive (neuromeningeal, maternal-fetal) listeriosis. Recently described as L. monocytogenes “hypervirulent” clones, no distinctive bacterial trait has been identified so far that could account for the differential pathogenicity of these strains. Here, we discuss some preliminary observations in experimentally infected mice suggesting that serovar 4b hypervirulent strains may have a hitherto unrecognized capacity for prolonged *in vivo* survival. We propose the hypothesis that protracted survivability in primary infection foci in liver and spleen—the first target organs after intestinal translocation—may cause L. monocytogenes serovar 4b hypervirulent clones to have a higher probability of secondary dissemination to brain and placenta.

## Abstract (precis)

Although all isolates of the foodborne pathogen Listeria monocytogenes are considered to be pathogenic, epidemiological evidence indicates that certain serovar 4b lineages are more likely to cause severe invasive (neuromeningeal, maternal-fetal) listeriosis. Recently described as L. monocytogenes “hypervirulent” clones, no distinctive bacterial trait has been identified so far that could account for the differential pathogenicity of these strains.

## OPINION/HYPOTHESIS

Listeria monocytogenes is the causative agent of listeriosis, a foodborne infection with severe manifestations in people with weakened immunity, pregnant women, and newborn infants. Clinically, listeriosis ranges from mild disease with flu-like symptoms and diarrhea to life-threatening conditions such as bacteremia and infections of the brain or placenta (1–3). The latter two are characteristic of the invasive form of the disease and are, respectively, known as central nervous system (CNS) or neuromeningeal listeriosis, typically a meningoencephalitis, and maternofetal/neonatal (MFN) listeriosis, presenting as miscarriage, stillbirth, or neonatal sepsis (4). Listeriosis is of great concern to the food industry due to the frequent occurrence of outbreaks and the cost of product recalls and food-safety measures (5). An important issue is that regulatory authorities consider all L. monocytogenes strains pathogenic, whereas only a few genotypes cause most listeriosis cases (6–8). There is therefore a pressing need to better understand L. monocytogenes diversity and its relationship with pathogenicity in order to target food safety interventions only to products contaminated by hazardous strains. Recent findings from integrated analysis of L. monocytogenes population genetics and epidemiological/clinical data (9) (see below) make the time ripe to discuss some unpublished observations from our laboratory that may help guide further research into this topic.

## L. MONOCYTOGENES DIVERSITY AND VIRULENCE HETEROGENEITY

L. monocytogenes is a slow-evolving yet diverse species that can be grouped into 4 major evolutionary lineages (I to IV), 13 lineage-related serovars (sv), and >100 clonal complexes (CC) defined by multilocus sequence typing (MLST) and whole-genome phylogenetic analysis (6, 10–14). While all strains of the species are potentially pathogenic, a wealth of epidemiological evidence indicates that it is pathogenically heterogeneous. Thus, only 3 of the 13 L. monocytogenes serovars, i.e., 4b and 1/2b within lineage I, and 1/2a within lineage II, are implicated in over 95% of human listeriosis cases (1, 2, 15). Comparative analyses of isolates from food surveys and clinical specimens (human or animal) also demonstrate an uneven distribution, with lineage II strains predominating in the former (chiefly sv 1/2a and sv 1/2c) and lineage I sv 4b strains in the latter (8, 16). Moreover, specific sv 4b clones, namely, CC1, CC2, CC4, and CC6, are overrepresented among clinical isolates and epidemic strains (9, 16), and tend to be isolated from patients with fewer or no immunocompromising comorbidities (9). At the other side of the spectrum, certain lineage II clones, such as CC9 and CC121, are strongly associated with a nonclinical (food) origin or, if causing infection, with highly immunocompromised patients (9). Consequently, the sv 4b CC1, CC2, CC4, and CC6 clones have been considered “hypervirulent,” the “food-associated” CC9 and CC121 “hypovirulent,” and the rest of the prevalent L. monocytogenes CCs “intermediate” (9). Interestingly, both CNS and MFN listeriosis are statistically associated with the hypervirulent L. monocytogenes clones, particularly, CC1 and CC4, in contrast to the hypovirulent clones CC9 or CC121, which are associated with bacteremia with no CNS or MFN involvement (9). Collectively, these observations support the notion that L. monocytogenes hypervirulent clones may possess specific attribute(s) that facilitate brain or placental infection.

## BASIS OF L. MONOCYTOGENES “HYPERVIRULENCE”: AN ELUSIVE QUESTION

L. monocytogenes hypovirulence has been linked to virulence gene polymorphisms, leading to attenuation (17, 18), notably mutations in the *inlA* gene which result in a truncated form of the invasion-associated protein InlA (9, 19). These *inlA* mutations are observed in 25 to 50% of lineage II food isolates and correlate experimentally with impaired entry into nonphagocytic cells (e.g., epithelial cells), offering a plausible explanation for the hypovirulent phenotype. On the other hand, pangenome studies have identified a number of accessory virulence-associated genes as specific to the hypervirulent (CC1, CC2, CC4, and CC6) clones (7, 9). Examples include the listeriolysin S gene cluster (LIPI-3) (20), sv 4b-specific teichoic acid biosynthetic genes (21), and a cellobiose family phosphotransferase system (PTS). Deletion of the latter has been reported to result in decreased CNS and fetal infection in mice (9), but it is only present in CC4 isolates, not in the other hypervirulent CCs. Other studies found two members of the internalin multigene family, InlF and Lmo2470 (InlP), to be involved in brain invasion (22) and placental tropism (23), respectively. However, both InlF and InlP are conserved across different L. monocytogenes lineages, inconsistent with a role in the differential pathogenicity exhibited by some sv 4b CCs. Whether any of the above described genetic determinants are actually mechanistically involved in L. monocytogenes tropism for brain and/or placenta requires additional investigation. To date, a clear differential functional marker that could be linked to L. monocytogenes “hypervirulence” (understood as an increased ability to cause invasive listeriosis) has not been identified.

## PROLONGED *IN VIVO* SURVIVAL OF HYPERVIRULENT SEROVAR 4b STRAINS

**FIG 1:**
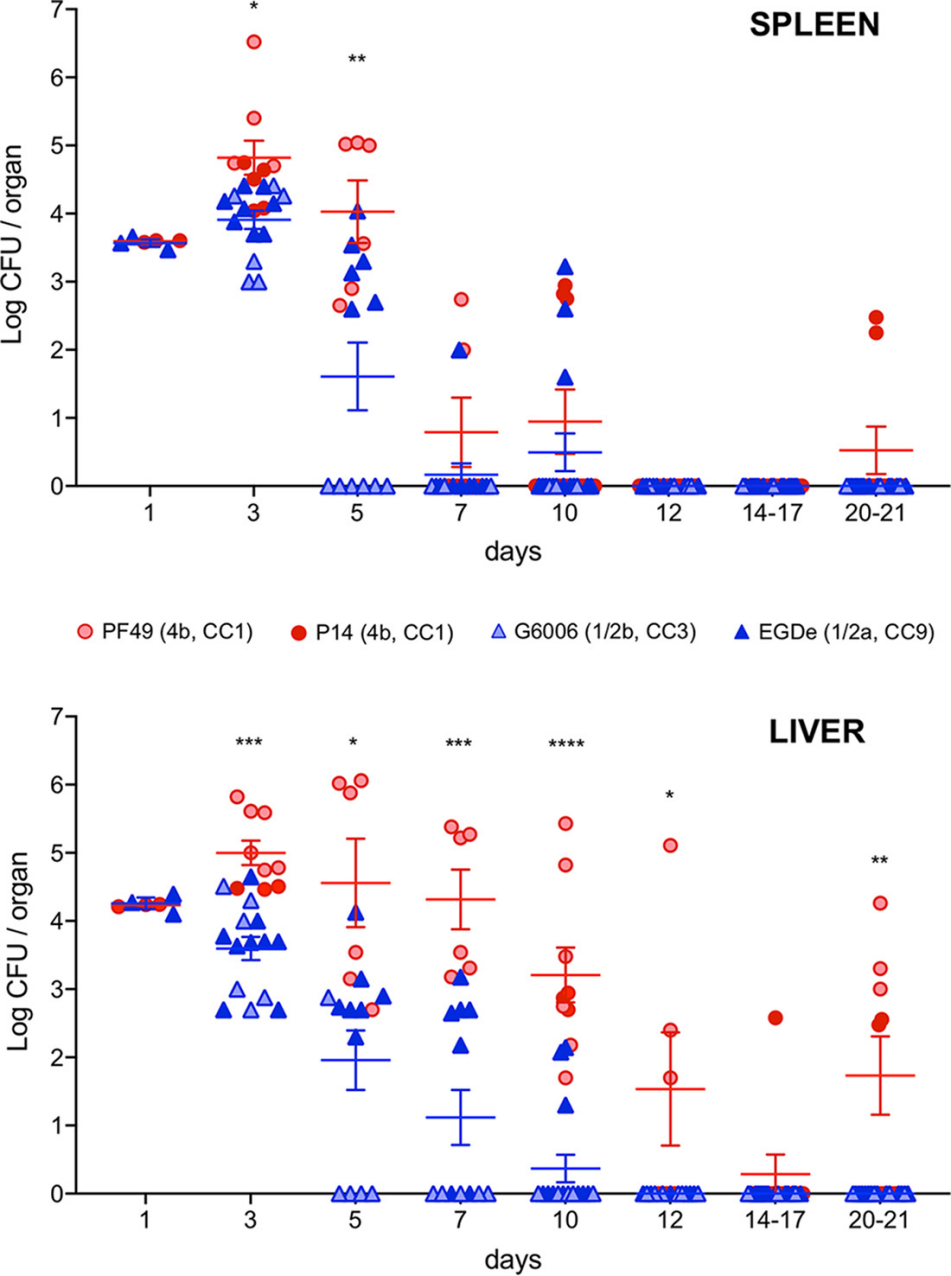
Prolonged *in vivo* survival of sv 4b (CC1) strains. Groups of 6- to 8-week-old BALB/c female mice (3 per group) were infected via the tail vein with a sublethal dose of *L. monocytogenes* (3 to 5 × 10^3^ CFU per animal). At the indicated time points, mice were euthanized, spleens and livers were recovered and homogenized, and bacterial numbers were determined by serial dilution and plate counting in brain heart infusion (BHI) agar. Experiments were performed at the Universidad Complutense de Madrid (two series with strains PF49, G6006, and EGDe) and the University of Edinburgh (additional series with strains P14 and EGDe). Each symbol represents a mouse. Data for mice infected with the sv 4b/CC1 strains PF49 and P14 are shown in red symbols; blue symbols represent those for strains G6006 and EGDe. Line diagrams in corresponding colors represent the combined mean ± standard error of the mean (SEM) for each of these two categories, with statistically significant differences indicated on top (two-way analysis of variance [ANOVA] and Fisher’s least significant difference test; *P* values: *, ≤0.05; **, ≤0.01; ***, ≤0.001; ****, ≤0.0001). Experiments were conducted according to applicable guidelines and regulations in animal experimentation (Complutense University: animal facility registration no. 28079-I5ABC-M, Real Decreto 223/1988, Orden 13/10/1989, EU Directive 86/609/CEE; Edinburgh University: UK Home Office project license under the 1986 Animals [Scientific Procedures] and approval by local Ethical Review Committee).

Preliminary data from mouse experiments in which we monitored listerial survival in organs beyond the typical standard 5- to 7-day time course, i.e., up to 20/21 days postinfection (p.i.), may offer some clues (Fig. 1). In these experiments, BALB/c mice were infected intravenously (i.v.) with four different L. monocytogenes isolates (Table 1). (i) PF49 is the epidemic strain of a cheese-associated outbreak in Switzerland where 79% of cases were CNS infections (24). (ii) P14 was isolated from an adult patient with CNS manifestations during a listeriosis outbreak in Spain (25). Both P14 and PF49 belong to the sv 4b hypervirulent clonal complex CC1. (iii) G6006 of sv 1/2b was responsible for an outbreak of febrile gastroenteritis due to chocolate milk in the United States, where none of the 45 affected people developed invasive listeriosis (26). This same strain was recovered from additional cases in the community, most of which were also noninvasive infections (febrile gastroenteritis, *n *= 5; bacteremia, *n *= 2; only one CNS infection, in a 72-year-old with several comorbidities) (26). G6006 belongs to CC3, which, comparatively, is much less frequently found among clinical isolates, is not statistically associated with invasive listeriosis, and is classified in the “intermediate virulence” category (9). Finally, (iv) the reference genome strain EGDe (27), of sv 1/2a, is widely used as an experimental model in L. monocytogenes pathogenicity studies (28). EGDe was supposedly a derivative of the sv 1/2a EGD strain used by Mackaness in his pioneering studies on cell-mediated immunity (29), in turn assumed to be one of the original isolates of E.G.D. Murray et al., who first identified L. monocytogenes in 1924 (30); however, EGDe was later shown to be genomically unrelated to EGD (28), and its origin is uncertain. EGDe belongs to the food-associated hypovirulent clone CC9, which is very rarely associated with clinical listeriosis (9). While EGDe exhibits the normal virulence features of L. monocytogenes in standard *in vitro* and *in vivo* experiments, it has been found to be poorly neuroinvasive in a mouse infection model (9). All four strains were confirmed to be wild type, including a wild-type *prfA* genotype with the usual virulence-related functional characteristics (31).

**TABLE 1:** L. monocytogenes strains

Strain^*a*^	Serovar	CC^*b*^	Source/description	Clinical manifestation	Reference
PF49	4b	CC1	Epidemic strain of cheese-associated outbreak, Vaud (Switzerland) 1983–1987	Neuromeningeal	24, 46
P14 (PAM 14)	4b	CC1	Listeriosis outbreak, Valencia (Spain) 1989	Neuromeningeal	25, 31, 47
G6006 (FSL-R2-0597)	1/2b	CC3	Epidemic strain of chocolate milk-associated outbreak, Illinois (USA) 1994	Noninvasive (febrile gastroenteritis)	26, 46
EGDe	1/2a	CC9	L. monocytogenes reference genome (T. Chakraborty)	Unknown	9, 27, 28

a Other designations in brackets.

b Clonal complex.

EGDe and G6006 displayed the expected behavior of L. monocytogenes in the organs of i.v. infected naive wild-type mice (Fig. 1). After a systemic infection, a progressive decrease in bacterial numbers is typically observed between days 3 and 7 until complete clearance by day 10 p.i. (32–35) as a consequence of effective macrophage activation and protective Th1 and CD8^+^ T-cell responses (36, 37). A similar pattern was exhibited by the sv 4b strains up to day 10 p.i., albeit with generally higher bacterial numbers, particularly in the liver. Strikingly, however, after virtual disappearance by day 14/17 p.i., the sv 4b bacteria were again recovered in significant numbers at day 20 or 21 for both PF49 and P14 in the liver and P14 in the spleen (Fig. 1).

The fact that both neurolisteriosis-associated isolates, PF49 and P14, exhibited the same behavior suggests that a capacity for prolonged *in vivo* survival might be a distinctive feature of the hypervirulent sv 4b strains compared to other L. monocytogenes genotypes. This ability has so far remained unnoticed because L. monocytogenes virulence studies have been historically (and currently still are) based on model strains of sv 1/2a such as EGDe or 10403S (28). Based on the abundant literature with sv 1/2a model strains, listerial full clearance from liver and spleen 7 to 10 days p.i. is the accepted “dogma” in wild-type mice systemically (i.v.) infected with a sublethal dose. Accordingly, most *in vivo* mouse studies with L. monocytogenes are generally limited to short infection time courses up to 5 to 7 days long (see, e.g., references 9 and 38 for recent examples).

## IMPLICATIONS FOR PATHOGENESIS

**FIG 2:**
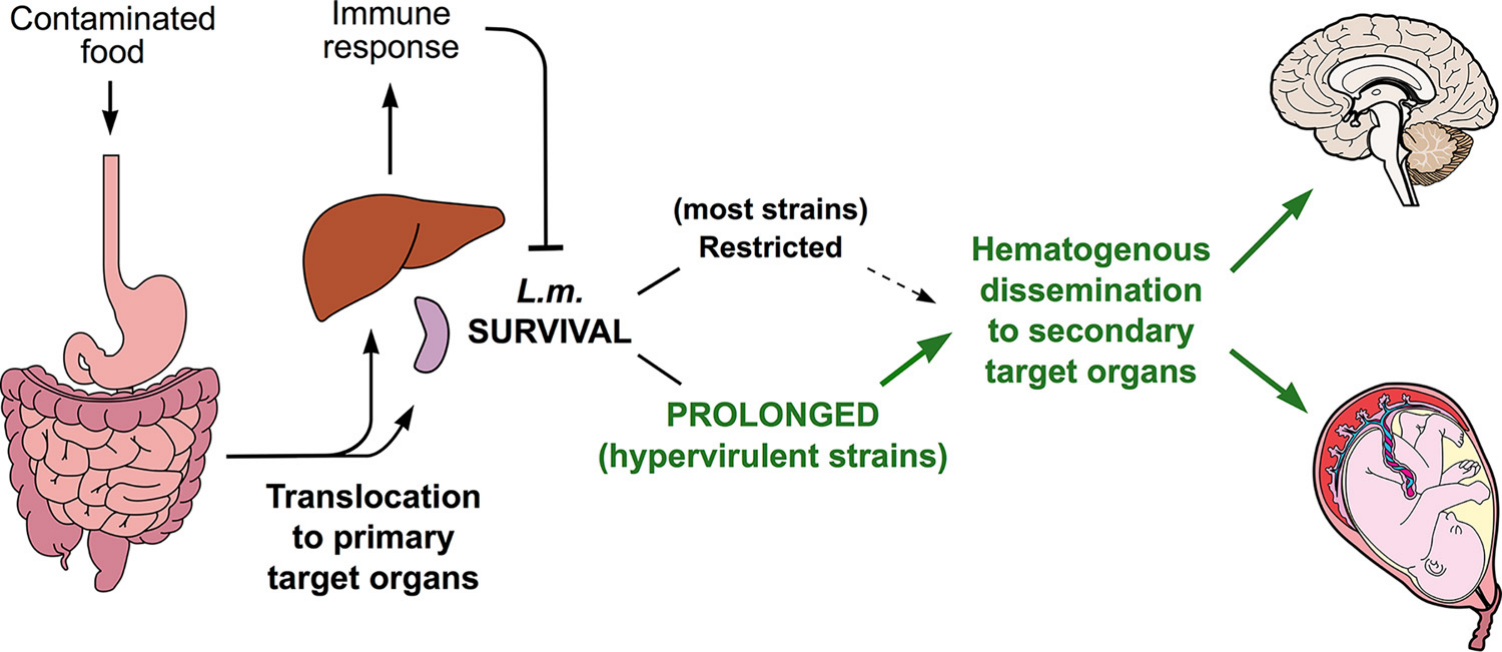
Model illustrating the hypothesis that prolonged survivability in primary infection foci in liver and spleen may explain the increased likelihood of L. monocytogenes serovar 4b hypervirulent strains to cause brain and placental infection. Schematic of the pathophysiology of invasive listeriosis modified from the original diagram in reference 40; see explanations therein and in reference 1 for details.

In the context of our pathophysiological understanding of listeriosis (1) (Fig. 2), prolonged *in vivo* survivability affords a reasonable explanation for why certain L. monocytogenes strains are more often associated with CNS or MFN infections. Listeriosis begins with bacterial crossing of the intestinal barrier and translocation to the primary target organs, i.e., the liver and spleen (1) (Fig. 2). In immunocompetent individuals, these initial stages are generally subclinical and self-limiting (unless a high L. monocytogenes dose is ingested, in which case febrile gastroenteritis may develop a few hours after ingestion of the contaminated food [39]). However, inadequate containment of the primary infection foci results in bacterial release into the bloodstream (bacteremia is, indeed, often observed in the course of listeriosis [4]) and dissemination of L. monocytogenes to the secondary target organs, i.e., the brain in immunocompromised adults or elderly people and the placenta in pregnant women (1, 40) (Fig. 2). Except for the ascending intra-axonal invasion of the rhombencephalon from oropharyngeal cranial nerve terminals, evoked in ruminants and occasionally in people (1, 41), neurolisteriosis generally results from hematogenous invasion of the brain (42, 43). In systemically infected mice, listerial brain invasion has been shown to critically depend on the level and duration of bacteremia (35). Studies of systemically infected pregnant guinea pigs also concluded that MFN listeriosis results from small numbers of L. monocytogenes bacteria trafficking from the maternal organs to the placenta (44). It can therefore be safely assumed that an ability for sustained survival at the primary infection sites in liver and spleen can directly translate into an increased likelihood of successful secondary dissemination of L. monocytogenes to the CNS or placenta (Fig. 2). This notion is consistent with the relatively long incubation period of CNS and MFN listeriosis, up to 14 to 67 days (45), which implies that invasive listeriosis clearly involves a protracted host-pathogen interaction process requiring prolonged bacterial survival.

## CONCLUDING REMARKS

We provide here an initial insight into a previously unrecognized differential virulence phenotype that offers a working hypothesis about why L. monocytogenes hypervirulent CCs may be more commonly associated with invasive listeriosis (Fig. 2). Further investigations should aim at systematically comparing the *in vivo* behavior of hypervirulent, hypovirulent, and intermediate CC strains (9), and ascertaining whether prolonged survival in primary infection foci in the liver and spleen results in increased hematogenous spread to brain and placenta. Our experiments were limited to a time course of 20/21 days, and it would be important to determine the duration of the *in vivo* survivability of L. monocytogenes and its relationship with bacteremia. During listeriosis, bacteremia occurs with or without CNS or placental infection; indeed, it is the clinical manifestation most commonly seen with hypovirulent CCs (9). Since hypovirulent CCs are typically found in highly immunocompromised patients or those with significant comorbidities (9), the association of these CCs with bacteremia may simply be a reflection of the early application of diagnostic blood cultures (systematically performed whenever a febrile process is detected in this vulnerable patient cohort) before invasive (typically brain) listeriosis can develop. Alternatively, hypervirulent strains could possess specific attributes, in addition to a prolonged *in vivo* survivability, that would promote brain and/or placental invasion. Further research should determine whether the hypervirulence of sv 4b CCs involves the presence/absence (or differential expression) of specific bacterial genetic determinants, as well as potential mechanisms of immune evasion or manipulation of host responses.
